# Mutation Landscape of Homologous Recombination Repair Genes in Epithelial Ovarian Cancer in China and Its Relationship With Clinicopathlological Characteristics

**DOI:** 10.3389/fonc.2022.709645

**Published:** 2022-02-03

**Authors:** Qianlan Yao, Yanhui Liu, Lihua Zhang, Lin Dong, Longlong Bao, Qianming Bai, Qian Cui, Jie Xu, Min Li, Jing Liu, Shannon Chuai, Jianming Ying, Zhihong Zhang, Xiaoyan Zhou

**Affiliations:** ^1^ Department of Pathology, Fudan University Shanghai Cancer Center, Shanghai, China; ^2^ Department of Oncology, Shanghai Medical College, Fudan University, Shanghai, China; ^3^ Institute of Pathology, Fudan University, Shanghai, China; ^4^ Department of Pathology, Guangdong Provincial People’s Hospital, Guangdong Academy of Medical Sciences, Guangzhou, China; ^5^ Department of Pathology, Southeast University, Zhongda Hospital, Nanjing, China; ^6^ Department of Pathology, National Cancer Center/National Clinical Research Center for Cancer/Cancer Hospital, Chinese Academy of Medical Sciences and Peking Union Medical College, Beijing, China; ^7^ Burning Rock Biotech, Guangzhou, China; ^8^ Department of Pathology, The First Affiliated Hospital of Nanjing Medical University, Nanjing, China

**Keywords:** homologous recombination repair gene, mutation, survival, next-generation sequencing, ovarian cancer

## Abstract

**Objective:**

The status of homologous recombination repair (HRR) gene mutations and their impact on the survival of patients with Chinese epithelial ovarian cancer (EOC) are still unclear. In this study, we retrospectively analyzed the mutations of HRR genes in tumor tissues and evaluated their values for predicting the survival of Chinese EOC patients.

**Methods:**

A total of 273 primary EOC patients from five different hospitals between 2015 and 2016 were recruited. All patients received staging surgeries or debulking surgeries combined with systemic platinum-based chemotherapy. DNA was extracted from formalin-fixed, paraffin-embedded sections and analyzed for mutations using a 21-gene panel (including 13 well-known HRR genes) by next-generation sequencing.

**Results:**

High-grade serous carcinoma (HGSOC) accounted for 76.2% of the cohort. A total of 34.1% (93/273) cases had 99 deleterious mutations in 9 HRR genes, namely, *BRCA1* (56/273, 20.5%), *BRCA2* (20/273, 7.3%), *ATM* (5/273, 1.8%), *RAD51C* (5/273, 1.8%), *RAD51D* (5/273, 1.8%), *BRIP1* (2/273, 1.8%), *CHEK2* (2/273, 0.7%), *FANCI* (2/273, 0.7%), and RAD*54L* (1/273, 0.4%). There is a strong mutual exclusion between HRR genes. The mutation landscape revealed several unappreciated deleterious variants in *BRCA1/2* and other HRR genes reported previously. Estimated according to the mutation allele frequency, about 4.8% of the patients had potential somatic HRR gene mutations, which might be underestimated. Moreover, HRR mutations mainly exist in HGSOC (83/208, 39.9%), clear cell (2/30, 6.7%), and endometroid subtypes (8/20, 40%), but not seen in other rare subtypes. *BRCA1* mutations tend to be present in younger patients with family history or multiple primary foci. Patients with *BRCA1/2* mutations tend to have a longer progression-free survival and overall survival, while other HRR mutation carriers tend to have a shorter progression-free survival, but no significant difference in overall survival.

**Conclusion:**

This study revealed the distribution of HRR gene mutations in Chinese EOC tissues. *BRCA1/2* account for the majority of HRR gene mutations and predict long prognosis in HGSOC. Non-BRCA HRR mutations also account for a very important proportion and might be associated with poor prognosis in HGSOC. It is suggested that HRR gene mutations need to be detected in EOC tissues and germline status be further clarified in clinical algorithm for potential targeted therapy, genetic screening, and prognosis prediction.

## Introduction

Epithelial ovarian cancer (EOC) is the third most common gynecological cancer among women in the world (1, 2) and the fourth most common in China (3). It is estimated that there were 52,100 new EOC cases and 22,500 EOC-associated deaths in China in 2015 (4). About 80% of EOC patients are diagnosed at late stage (III and IV) and 5-year survival rates are less than 30% (5). The overall 5-year survival of EOC ranges between 30% and 40% worldwide and has slightly increased by 2%~4% over the last two decades (6). Moreover, 70% of patients with advanced epithelial EOC will relapse, and the survival for the relapsed EOC is extremely low.

Homologous recombination repair (HRR) is an important pathway that allows the repair of double-stranded DNA breaks. Accumulated data have demonstrated that deficiency in HRR genes *BRCA1* and *BRCA2* accounts for the majority of familial EOC (7, 8). Further investigation highlights that successful HRR repair also requires multiple other protein co-factors, including *RAD51C*, *RAD51D*, *BRIP1*, *PALB2*, and *BARD1* (9, 10). Several publications have reported the presence of somatic *BRCA1/2* mutations in EOC, highlighting that both germline and somatic mutations in HRR genes can result in EOC (11). The Cancer Genome Atlas (TCGA) reported that HRR gene mutations exist in 50% of high-grade serous EOC. What is more, the incidence of HRR germline mutations in men with metastatic castration-resistant prostate cancer is 11%–33%, and the most common mutant HRR genes include *BRCA2*, *CDK12*, *ATM*, *CHEK2*, *BRCA1*, *MSH2*, *FANCA*, *MLH1*, and *RAD51* (12). Different histologic subtypes and races show different mutation rates in HRR genes in EOC (13); however, the distribution of HRR gene mutations and their correlation with clinicocharateristics in Chinese population are still not clear. It has been reported that *BRCA1/2*-associated EOC show improved overall survival (OS) and sensitivity to both platinum chemotherapy and PARPi (poly ADP-ribose polymerase inhibitors). PARPis such as niraparib, olaparib, and rucaparib have been approved by the Food and Drug Administration (FDA) and European Medicines Agency (EMA) in the maintenance setting for EOC patients who achieved a CR (complete response) or PR (Partial response) following platinum-based chemotherapy (14, 15). Several PARPis are at an early stage of clinical development and require more research, such as talazoparib (16). *In-vitro* studies have shown that defects in other HRR (non-BRCA HRR) genes (such as *ATM*, *CHEK1*, *CHEK2*, and *RAD51D*) also confer sensitivity to PARP inhibitors (17, 18). Furthermore, a subset of sporadic (BRCA wild-type) recurrent platinum-sensitive EOC showed sensitivity to PARP inhibitors (19), which might be attributable to the influence of undetected HRR gene alterations. PARP inhibitors as a treatment option for EOC and the possibility of genetic changes other than BRCA genes are currently under investigation (NCT-02476968, ORZORA study). However, other *in-vitro* studies suggest that no single HRR gene mutation shows perfect correlation with sensitivity to platinum and PARP inhibitor (20). In addition, it is well known that some HRR genes such as *BRCA1/2*, *ATM*, *BRAD1*, *BRIP1*, *PALB2*, *RAD51C*, and *RAD51D* are associated with high risk of ovarian cancers, and tissue detection might be a good way to learn the germline status.

There have been some prevalence studies on HRR genes in EOC. The mutation rates varied a lot in different study background, e.g., histological subtype, human race, and stage. Studies show that 5% to 29% of EOC patients harbored *BRCA1/2* mutations. In China, a multicenter clinical study showed that 28.5% of EOC patients had *BRCA1/2* germline mutation (21). A few studies reported that the prevalence of other HRR gene mutation ranges from 3% to 10% (22). These studies largely focused on patients from the white population and few from the Asian population. In China, the contribution of HRR gene mutation to EOC (especially tissue-derived EOC) has not yet been fully explored. Thus, it is important to understand the distribution of HRR gene mutations in EOC tissues and their association with clinical characteristics, and it will be very helpful for potential PARPi targeted therapy, further genetic screening, and prognosis prediction.

**Figure 1:**
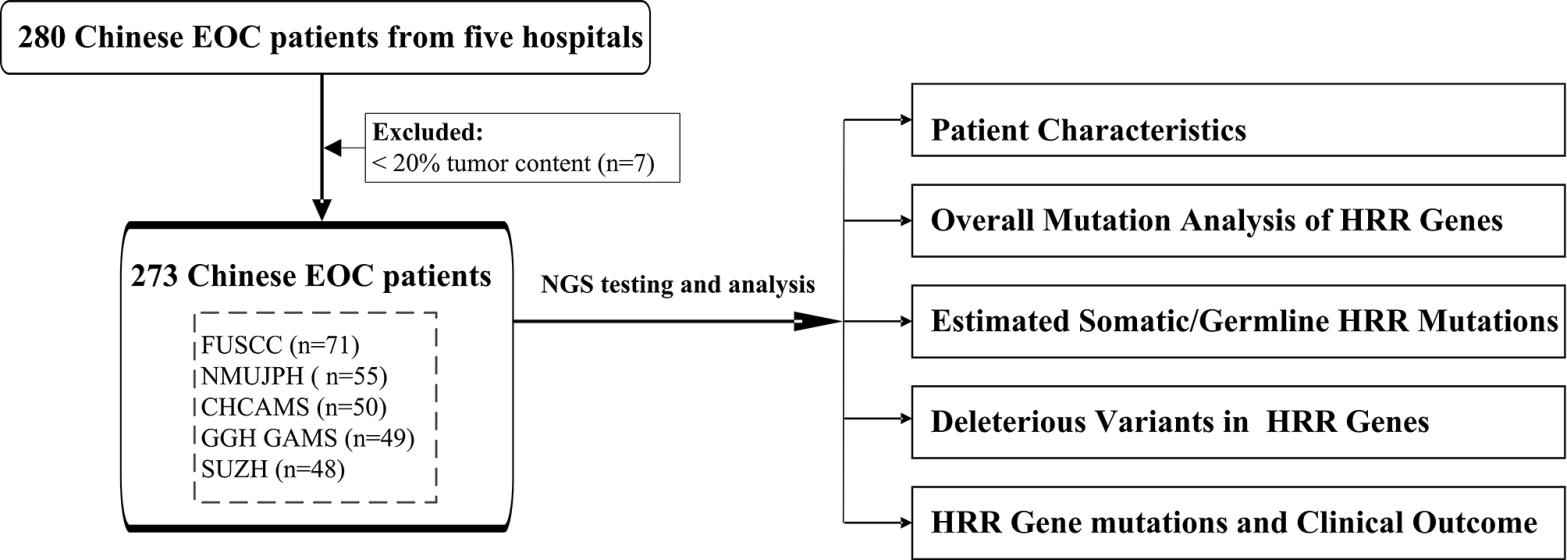
The workflow diagram of this study. FUSCC, Fudan University Shanghai Cancer Center; NMUJPH, The First Affiliated Hospital of Nanjing Medical University; CHCAMS, National Cancer Center/National Clinical Research Center for Cancer/Cancer Hospital, Chinese Academy of Medical Sciences and Peking Union Medical College; GGH GAMS, Guangdong Provincial People’s Hospital, Guangdong Academy of Medical Sciences; SUZH, Southeast University, Zhongda Hospital.

In this study, 273 unselected patients with EOC were enrolled from five different hospitals in China to comprehensively explore the HRR gene mutations in Chinese population. We applied targeted next-generation sequencing (NGS) with 21 genes (including 13 HRR genes and 8 non-HRR genes) using tissue formalin-fixed paraffin-embedded (FFPE) samples. The clinical characteristics of HRR mutation carriers were also assessed. The workflow diagram is shown in **Figure 1**. A multicenter study was conducted in order to better reflect EOC patients from different regions of China and to promote the detection ability of NGS at the Department of Pathology in the local hospital.

## Materials and Methods

### Study Population and Clinical Data Collection

A total of 280 patients diagnosed with EOC in the years 2015–2016 were collected from five hospitals. After excluding 7 unqualified samples with tumor content less than 20%, 273 patients were included in this study from Fudan University Shanghai Cancer Center (FUSCC; 71 cases), The First Affiliated Hospital of Nanjing Medical University (NMUJPH; 55 cases), National Cancer Center/National Clinical Research Center for Cancer/Cancer Hospital, Chinese Academy of Medical Sciences and Peking Union Medical College (CHCAMS; 50 cases), Guangdong Provincial People’s Hospital, Guangdong Academy of Medical Sciences (GGH GAMS; 49 cases), and Southeast University, Zhongda Hospital (SUZH; 48 cases). Patients were enrolled at diagnosis and were not selected by age, familial cancer history, or histological subtype. The project was approved by the Institutional Review Board of the hospitals. Tumor histology was confirmed by two independent pathologists. Clinical and pathological information was extracted from the database of the institutional patient, including age at diagnosis, tumor histopathology, stage (International Federation of Gynecology and Obstetrics), and personal and family history of cancer when available. Hereditary breast and ovarian cancer (HBOC) syndrome-related tumors were defined as breast, ovarian, and pancreatic cancers in women and cancers of the breast and prostate in men. Samples with incomplete or undetermined information were described as “unknown”.

### DNA Sequencing and Variants Calling

Genomic DNA (gDNA) was extracted from FFPE sections in area with a minimum neoplastic cellularity of 20%. DNA quantification, library construction, hybridization, and massively parallel sequencing were performed in each center using Burning Rock HRDv1 panel (Burning Rock Company, China). Firstly, gDNA was extracted and quantified using Qubit and NanoDrop. Then, gDNA was randomly fragmented by Covaris. After 2 rounds of bead purification, gDNA fragments were mainly distributed between 200 and 400 bp. AdA adaptor-ligase was used for ligation of DNA fragments with blunt and single base overhang, and the AdA adaptor-ligated fragments were amplified by polymerase chain reaction (PCR). Next, the PCR products were used for follow-up exon capture. The captured fragments were subsequently purified, amplified, ligated with AdB, and circularized. Finally, high-throughput sequencing of library products was performed by Illumina MISeq sequencing. All exons of each target gene were sequenced. A total of 21 genes were designed in this panel, including 13 well-known HRR-related genes (*BRCA1*, *BRCA2*, *ATM*, *BRIP1*, *PALB2*, *RAD51C*, *BARD1*, *CHEK1*, *CHEK2*, *RAD51B*, *RAD51D*, *RAD54L*, *FANCI*) and other 8 non-HRR genes (*ATR*, *EMSY*, *FAM175A*, *FANCA*, *MRE11A*, *NBN*, *PTEN*, *RAD50*) (23). All coding regions and exon–intron boundaries ( ± 20 bp) of HRR genes were screened. All reads from the prepared libraries that passed the Illumina Chastity filter were formatted into fastq files. The fastq files were aligned to the genome using BWA (v.0.7.10) (24) against the human genome build version 19. BAM files generated from alignment reads were preprocessed using GATK v.3.2 (25). Point mutation and small indels were identified by MuTect algorithm2 (http://www.broadinstitute.org/cancer/cga/MuTect) and Varscan2 v.2.4.3 (26). Large rearrangements and copy number variants were identified using VarDict and domestically developed suite software. Filtered point mutations and indels were annotated using SnpEff and ANNOVAR. Variants were named according to HGVS (Human Genome Variation Society; http://www.hgvs.org/) nomenclature and interpreted by two independent pathologists into five classes (benign, likely benign, uncertain significance, likely pathogenic, pathogenic) in accordance with the principles published in the ACMG guideline (27).

### Statistical Analysis

Chi-square test was used to analyze contingency tables. The Kruskal–Wallis test was used to test the relationship between one nominal variable and one continuous variable. Patients with specific missing clinical data were not included in relevant specific clinical characteristic analyses. The observation time for OS ranged from the date of the first surgery to the date of death or the study end date/last follow-up date, whichever occurred first. The endpoint for progression-free survival (PFS) was either the date of first recurrence or the last follow-up, starting from the completion of frontline chemotherapy. Kaplan–Meier survival analysis was performed and statistical significance was assessed using the log-rank test. Cox analysis was used to adjust the *p*-value by age, family history/multiple primary-related malignancy information (multiple primary foci), and pathologic stage. A *p*-value <0.05 was considered statistically significant. All the calculations were conducted with functions provided in R (https://www.r-project.org/).

Mutual exclusion between HRR genes were compared using Fisher’s exact test. An estimate of the odds ratio (OR) >5 denotes a higher likelihood of co-occurrence, and OR <0.5 denotes a higher likelihood of mutual exclusivity. *p <*0.05 was considered as statistically significant.

## Results

### Patient Characteristics

After excluding 7 unqualified patients, 273 patients diagnosed as EOC were included in this study. **Table 1** depicts the major clinicopathological characteristics of the study subjects. Median age at diagnosis in our cohort was 53 years old (ranging from 21 to 87). Patients over 50 years old accounted for 60.1%, and those below 50 years old accounted for 39.9%. High-grade serous carcinoma (HGSOC) accounted for the majority of our cohort (208, 76.2%), and there are 30 (11.0%) clear cell subtype, 20 (7.3%) endometroid subtype, and 15 (5.5%) other cases [including 2 low-grade serous subtype tumors (LGSOC), 4 sarcomatoid, and 2 serous borderline tumors]. Most cases (173; 63.4%) were stage III. A total of 48 (17.6%) cases have family history and 22 (8.1%) cases have multiple primary tumors.

**Table 1:** The major clinicopathological characteristics, *BRCA1/2*, and other HRR gene mutations in epithelial ovarian cancers.

Characteristics	No. of patients (%)	*BRCA1*m (%)	*BRCA2*m (%)	Other HRRm (%)	*p*-value
**Age**					
[median (min, max)]: 53 (21, 87)	273 (100)	56 (20.5)	19 (6.9)	18 (6.5)	
<=50	109 (39.9)	29 (26.7)	4 (3.7)	8 (7.3)	0.065
>50	164 (60.1)	27 (16.5)	15 (9.1)	10 (6.1)	
**Histology**					
High-grade serous carcinoma	208 (76.2)	53 (25.5)	18 (8.7)	12 (5.8)	0.009
Clear cell	30 (11.0)	0 (0)	0 (0)	2 (6.7)	
Endometroid	20 (7.3)	3 (15)	1 (5)	4 (20)	
Other/unknown	15 (5.5)	0 (0)	0 (0)	0 (0)	
**Stage**					
I	43 (15.8)	3 (7.0)	0 (0)	7 (16.3)	0.005
II	24 (8.8)	7 (29.1)	1 (4.2)	1 (4.2)	
III	173 (63.4)	37 (21.4)	15 (8.7)	10 (5.8)	
IV	33 (12.1)	9 (27.3)	3 (9.1)	0 (0)	
**Family history**					
N	225 (82.4)	40 (17.8)	14 (6.2)	15 (6.7)	0.639
Y	48 (17.6)	16 (33.3)	5 (10.4)	3 (6.3)	
**Tumor history/multiple primary foci**					
N	251 (91.9)	47 (18.7)	17 (6.8)	16 (6.4)	0.919
Y	22 (8.1)	9 (40.9)	2 (9.1)	2 (9.1)	

### Overall Mutation Analysis of HRR Genes

**Figure 2:**
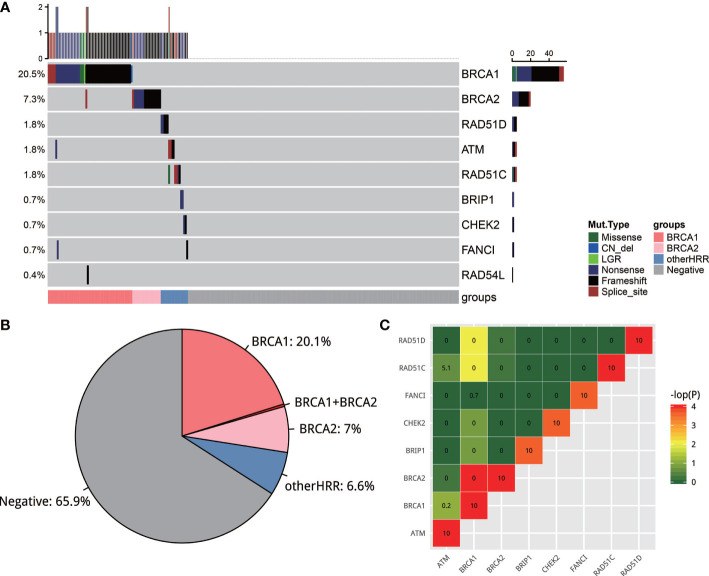
**(A)** The mutation landscape of homologous recombination repair (HRR) gene in EOC. Seven of the 13 well-known HRR genes were mutated. **(B)** Pie plot of the distribution of carriers of HRR genes. **(C)** Strong mutual exclusion between HR genes. The closer the color is to green or the smaller the number is, the more mutually exclusive the gene mutations are. The closer the color is to red or the larger the number is, the more mutually exclusive the gene mutations are.

Two hundred and seventy-three of 280 samples from five hospitals successfully passed the NGS quality control, with a median depth of 1,329 and median depth of 661 after reduplication. Variants were classified into five classes based on the ACMG guideline (27) and those pathogenic/likely pathogenic (deleterious variants, defined as mutation) were used for further analysis. A total of 34.1% (93/273) cases had 99 deleterious mutations in 9 HRR genes (**Figure 2A**). These 9 HRR genes are *BRCA1*, *BRCA2*, *ATM*, *BRIP1*, *CHEK2*, *FANCI*, *RAD51C*, *RAD51D*, and *RAD54L*. A total of 27.6% (75/273) cases had *BRCA1*/*BRCA2* mutations and 6.6% of the cases (18/273) only had other HRR (non-*BRCA1/2* HRR) mutations (**Figure 2B**). More specifically, *BRCA1/2* mutations were observed most frequently: *BRCA1* mutations occurred in 20.5% (56/273) of the cases, and *BRCA2* occurred in 7.3% (20/273) of the cases, which was consistent with the previous observation (21, 28). *ATM*, *RAD51C*, and *RAD51D* had a mutation frequency of 1.8% (5/273). *BRIP1*, *CHEK2*, and *FANCI* had a mutation frequency of 0.7% (2/273) and *RAD54L* 0.4% (1/273). Among 273 cases, only 6 patients had more than one HRR mutations. Of these 6 patients, 4 harbored both *BRCA1/2* and other HRR gene mutations, 1 harbored both *BRCA1* and *BRCA2* mutation, and 1 harbored two *BRCA2* mutations. In non-HRR genes, *PTEN* mutations occurred with the highest frequencies (14/273; 5.1%) (**Figure S1**). The mutation rates in five hospitals were slightly but not significantly different (chi-square test; *p* = 0.368). The frequency of *BRCA1/2* carriers ranged from 19% to 36%, while the frequency of HRR carriers ranged from 25% to 44% in the five hospitals. All variants found in this study could be found in **Supplementary Table 1**.

It is interesting to note that there was a strong mutual exclusion between HRR genes. To test this, exclusive score OR was used to depict this phenomenon: OR >5 tends to co-occur, and OR <0.5 tends to be mutually exclusive. As shown in **Figure 2C**, HRR genes exhibit universal mutual exclusion. For example, *BRCA1* is significantly exclusive with *BRCA2*, *RAD51C*, and *RAD51D* (*p* < 0.05). We also noticed that non-HRR genes *NBN*, *PTEN*, and *MRE11A* always co-occurred with HRR mutations (**Figure S1C**).

### Estimated Somatic and Germline HRR Mutations

Clinical trials showed that EOC patients with both gBRCA (germline *BRCA1/2*) and sBRCA (somatic *BRCA1/2*) could benefit from platinum-containing agents and PARP inhibitors (poly[ADP-ribose] polymerase) (29, 30). Tissue detection could identify tumor mutations including both gHRR (germline HRR) and sHRR (somatic HRR) mutations. So, we next assessed the potential proportion of sHRR mutations in 99 deleterious mutation carriers in tissues. Due to the lack of gHRR gene detection, a mutation with an allele frequency (AF) value less than 0.3 and a mutation with a CNV loss were estimated as potential sHRR mutations. Most cases (81/93; 87.1%) were detected as potential gHRR mutations (**Table 2**). *BRCA1*, *BRCA2*, *ATM*, *RAD51C*, and *RAD51D* were the most recurrent germline mutations, accounting for 53.5% (53/99), 17.2% (17/99), 2% (2/99), 5.1% (5/99), and 5.1% (5/99) in 99 HRR deleterious mutation carriers, respectively (**Table 3**). *BRCA1/2* are the most recurrent somatic mutations, accounting for 7% in 99 HRR deleterious mutation carriers. More importantly, 13 cases with 14 mutations were estimated to have potential sHRR (**Table 2**), accounting for 13.1% (13/99) of the deleterious mutation carriers and 4.8% (13/273) of all EOC samples. Among them, 1 patient was inferred to have both germline (c.1185G>A p.Trp395*, AF = 40%) and somatic (c.2710G>T p.Gly904*, AF = 17%) *BRCA2* mutation. When focusing on *BRCA1/2*, 2.6% (7/273) EOC and 2.8% (6/208) HGSOC samples had potential somatic mutations, which were much lower than those of a recent study (7.1%) (31) and our previous study (8.7%) (32).

**Table 2:** Estimated proportion of germline/somatic HRR mutations.

	Criteria	No. of mutations (%)	No. of patients (%)
**Potential germline mutation**	AF >= 30%	86 (86.9%)	81 (87.1%)
**Potential somatic mutation**	AF < 30%/CNV del	13 (13.1%)	13 (14.0%)

**Table 3:** Statistics on the prevalence of estimated germline/somatic mutations among 99 deleterious mutation in each HRR gene.

Gene	% (no.) P/LP mutation		
	All	Germline	Somatic
*BRCA1*	56.1% (56)	53.5% (53)	3% (3)
*BRCA2*	21.2% (21)	17.2% (17)	4% (4)
*ATM*	5.1% (5)	2% (2)	3% (3)
*RAD51C*	5.1% (5)	5.1% (5)	0
*RAD51D*	5.1% (5)	5.1% (5)	0
*BRIP1*	2% (2)	1% (1)	1% (1)
*CHEK2*	2% (2)	1% (1)	1% (1)
*FANCI*	2% (2)	1% (1)	1% (1)
*RAD54L*	1% (1)	1% (1)	0
All	99	86.9% (86)	13.1% (13)

### Deleterious Variants in *BRCA1/2* and Other HRR Genes

**Figure 3:**
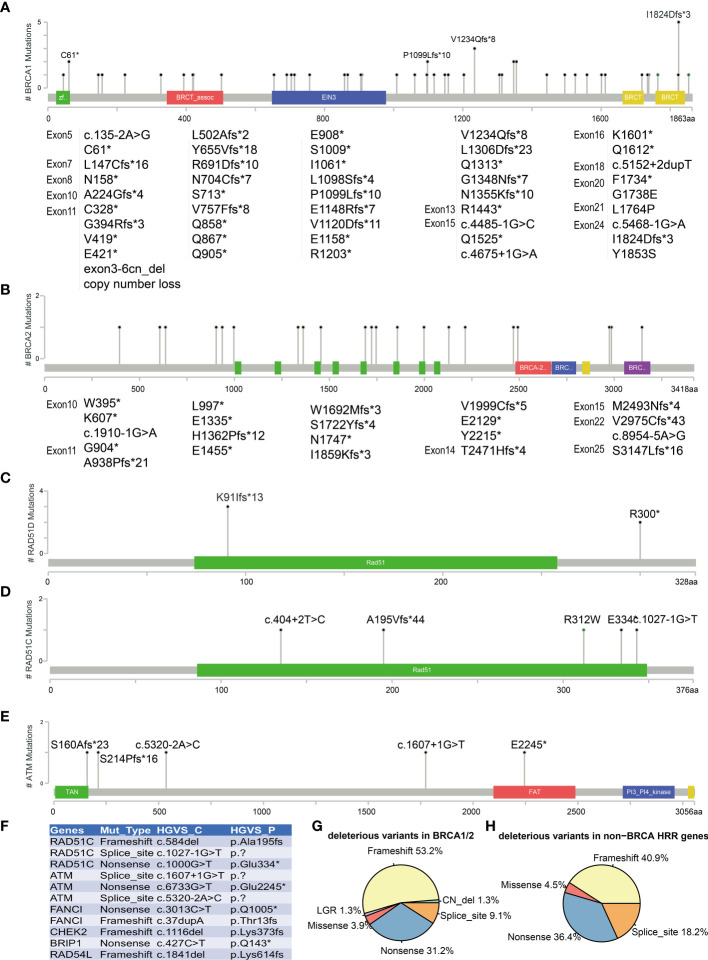
Mutations landscape of *BRCA1* **(A)**, *BRCA2* **(B)**, *RAD51D* **(C)**, *RAD51C* **(D)**, and *ATM* **(E)** genes. The *X*-axis represents the amino acid residues of the proteins, and the *Y*-axis represents the frequencies of each type of mutations. **(F)** List of 11 novel non-BRCA HRR variants. Distribution of mutation types in *BRCA1/2* **(G)** and non-BRCA HRR genes **(H)**. The symbol * means the variant is a nonsense mutation in HGVS.

All *BRCA1/2* and other HRR gene mutations were scattered throughout the whole gene, without hotspot mutations. **Figures 3A–E** show the five most frequently mutated HRR genes. Of the 77 mutations identified in *BRCA1/2*, 53.8% had frameshift mutations, 30.8% had nonsense mutations, 9% had splice site mutation, 3.8% had missense mutations, and 2.6% had copy number loss (**Figure 3G**). Six mutations were observed in more than two cases in *BRCA1* (c.183T>A C61*, c.3294del P1099fs, c.3700_3704del V1234fs, c.4041_4042del G1348fs, c.4065_4068del Asn1355fs, c.5470_5477del I1824fs). The most common *BRCA1* mutation detected in this study was c.5470_5477del (p.Ile1824fs), which has been documented by ClinVar and seemed to be specific to Asian ethnicity (33). In addition, we also found four novel mutations that have not been reported by ClinVar (https://www.ncbi.nlm.nih.gov/clinvar) and the BRCA Exchange database (https://brcaexchange.org/), consisting of one nonsense mutation (c.3026C>G p.S1009*) and three frameshift mutations (c.5201_5202del p.F1734fs, c.471dupT p.N158fs, and c.1179dup p.G394fs). Of the 21 unique mutations identified in *BRCA2*, none of them was observed in more than one case. Four novel *BRCA2* mutations that were not reported in ClinVar and BRCA Exchange may only exist in Chinese population (c.9439del p.S3147fs, c.6645C>A p.Y2215*, c.8922dupT p.V2975fs, and c.7477dupA p.M2493fs). In addition, two cases carried two deleterious BRCA mutations (AL1700132FFP: *BRCA2* c.2710G>T p.G904*, AF = 17%, and *BRCA2* c.1185G>A p.W395*, AF = 40%; RS1724106FFP: *BRCA1* c.2071del p.R691fs, AF = 77%, and *BRCA2* c.8954-5A>G, AF = 36%). Twenty-two (19 unique) non-BRCA HRR mutations were also detected in this study. *ATM*, *RAD51C*, and *RAD51D* are the most frequently mutated non-BRCA HRR genes with a mutation frequency of 1.8% (5/273) (**Figures 3C, D** **)**. These mutations included eight nonsense mutations, nine frameshift mutations, four splice sites, and one missense mutation (**Figure 3H**). Of these mutations, only two variants were recurrent and they were both harbored in *RAD51D* (c.270_271dup p.Lys91fs and c.898C>T p.Arg300*) (**Figure 3C**). Eleven novel non-BRCA HRR variants that were not reported by ClinVar were also observed in this study, including *RAD51C* (c.584del p.Ala195fs, c.1027-1G>T and c.1000G>T p.Glu334*), *ATM* (c.1607+1G>T, c.6733G>T p.Glu2245*, and c.5320-2A>C), *FANCI* (c.3013C>T p.Gln1005* and c.37dupA p.Thr13fs), *CHEK2* (c.1116del p.Lys373fs), *BRIP1* (c.427C>T p.Gln143*), and *RAD54L* (c.1841del p.Lys614fs) (**Figure 3F**).

### Association Between HRR Gene Mutations and Clinical Outcome

**Figure 4:**
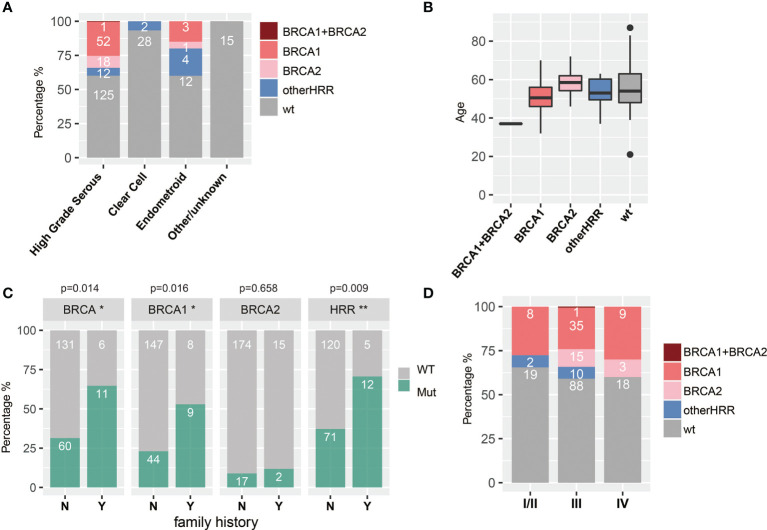
Associations between HRR mutations and histopathologic **(A)** subtypes, **(B)** age, **(C)** family history and multiple primary foci (Y means cases with family history or multiple primary foci, and N means none; * means significant difference between mutation of that gene and multiple primary foci or family history), and **(D)** stage.

HGSOC, clear cell subtype, and endometroid subtype are the major histopathologic subtypes in our study, with 208, 30, and 20 cases, respectively. HRR gene mutation frequency was up to 39.9% (83/208) and 40% (8/20) in HGSOC and endometroid subtypes, respectively, while there was only 6.7% (2/30) in the clear cell subtype. Moreover, HGSOC had more *BRCA1/2* mutations (34.1%, 71/208) than endometroid (20%, 4/20) (chi-square test; *p* = 0.225) and the clear cell subtype (0%) (chi-square test; *p* < 0.001), and 5.8% (12/208) of the HGSOC, 20% (4/20) of the endometroid, and 6.7% (2/30) of the clear cell subtype harbor non-BRCA HRR mutation. In addition, HRR mutations were not detected in two patients with LGSOC, four patients with sarcoma, and two patients with borderline serous tumors (**Table 1** and **Figure 4A**). Since HGSOC accounts for the vast majority (*n* = 208) of the subjects, the following analysis is specific for this subtype. These 208 cases were divided into five groups based on age (**Figure 4B**). The ages in the *BRCA2* carrier group are significantly greater than those in the *BRCA1* carrier group (*t*-test; *p* = 0.006), non-BRCA HRR group (*t*-test; *p* = 0.146), and wild-type group (*t*-test; *p* = 0.204). Specially, one case with both *BRCA1* and *BRCA2* had the youngest age in all groups. The onset at younger age would indicate the higher detection rate of *BRCA1* mutations. Patients with family history or multiple primary foci have a higher *BRCA1* mutation rate (chi-square test; *p* = 0.0016) and HRR mutation rate (chi-square test; *p* = 0.009) (**Figure 4C**). If only based on family history or multiple tumor features for detection, 71 patients with HRR mutations might be missed, accounting for 85.5% (71/83) of the cases with HRR mutations. There was no significant difference in HRR gene mutation rate between early stage (I and II) and late stage (III, IV) groups (**Figure 4D**).

**Figure 5:**
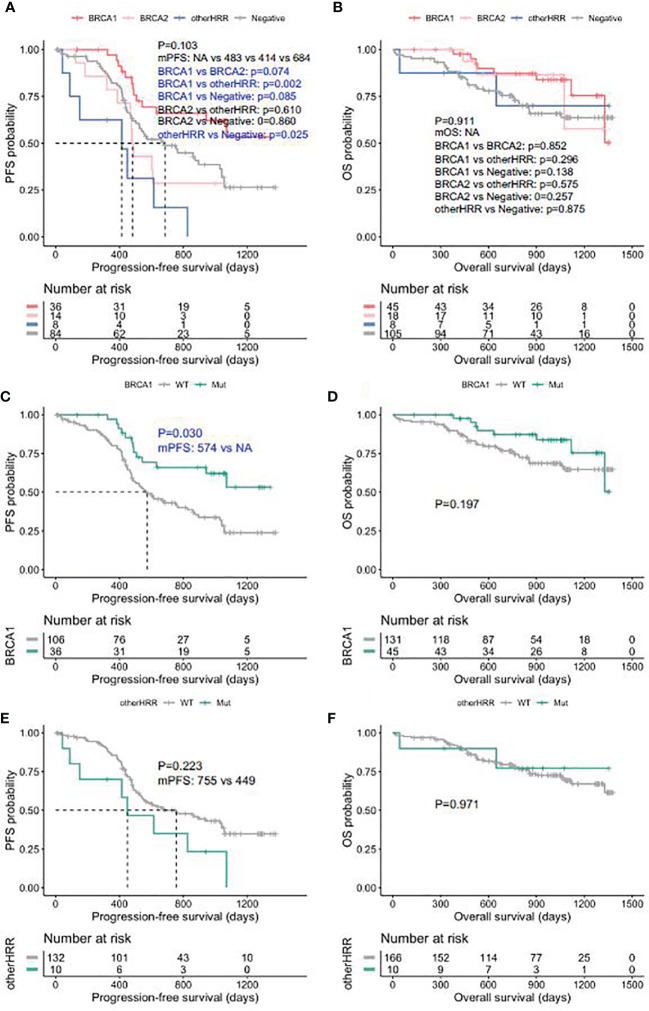
Progression-free survival (PFS) and overall survival (OS) in ovarian carcinoma patients by HRR mutation category. **(A)** PFS between deleterious *BRCA1*, *BRCA2*, non-BRCA HRR, and without HRR mutation carriers. **(B)** OS between deleterious *BRCA1*, *BRCA2*, non-BRCA HRR, and without HRR mutation carriers. **(C)** PFS with and without deleterious *BRCA1* mutations. **(D)** OS with and without deleterious *BRCA1* mutations. **(E)** PFS with and without non-BRCA HRR mutations. **(F)** OS with and without non-BRCA HRR mutations.

Patients with HGSOC who received platinum chemotherapy were followed up, and the median follow-up was 28.7 months (interquartile range: 16.3–36.4 months). *BRCA1* carriers had better PFS than HRRwt (HRR gene wild type) cases and *BRCA2* carriers (**Figure 5A**), while the PFS of other HRRm (HRR genes mutations) carriers (414 days) was significantly shorter than HRRwt cases (*p* = 0.025) (**Figure 5A**). Moreover, *BRCA1/2* carriers had a trend of better OS than HRRwt and non-BRCA HRRm carriers (**Figure 5B**). More specifically, the PFS of *BRCA1*m (*BRCA1* mutation) carriers was significantly longer than that of *BRCA1*wt (*BRCA1* wild type) carriers (*p*-value = 0.030) (**Figure 5C**). *BRCA1*m carriers had a trend of better OS than *BRCA1*wt carriers (**Figure 5D**). However, non-BRCA HRRm carriers had a trend of poorer PFS than HRRwt carriers (**Figure 5E**). There were no significant differences between the OS of non-BRCA HRR mutation carriers and HRRwt carriers (**Figure 5F**).

## Discussion

This retrospective analysis is performed to investigate the mutation profiles and clinicopathological features of tissue HRR genes in EOC patients using NGS in five hospitals in China. Our data show that about 40% ovarian patients had HRR mutations including germline or somatic mutations in tumor tissues. The prevalence rates of *BRCA1/2* and other HRR mutations are 27.6% and 6.6%, respectively. *ATM*, *RAD51C*, and *RAD51D* are the most frequently mutated genes among non-*BRCA1/2* HRR genes. This suggests that *BRCA1/2*-only screening may miss ~6.6% of the cases. A clinical trial has suggested that patients with HRRm but without BRCAm tumors might gain more benefit from olaparib compared with patients with no detectable HRRm in EOC (34). Olaparib has been approved by the FDA for HRR gene-mutated metastatic castration-resistant prostate cancer (23). Therefore, the HRR panel is suggested in ovarian cancer to obtain comprehensive HRR mutation information. Clinical trials showed that both germline and somatic HRR mutations might benefit from platinum-containing agents and PARP inhibitor (29, 30). There were 4.8% of cases harboring potential somatic HRR gene mutations in our cohort, of which 53.8% (7/13) were *BRCA1/2* somatic mutations. In addition, *BRCA1/2*, *ATM*, *BRIP1*, *PALB2*, *RAD51C*, and *RAD51D* gene mutations are reported to be associated with high risk of ovarian cancers, and from the results of tumor tissue, it is easy to further determine their genetic status. Based on all the above, we would recommend screening HRR gene alterations on tumor tissue and further verifying whether it is a germline variant or not through site-specific Sanger sequencing. From the study, we also found that HRR genes exhibit universal mutual exclusion with each other. This exclusive characteristic may be helpful in the annotation of variants. If a definitely pathogenic/likely pathogenic mutation is found in a patient, then other HRR gene variants are more likely to be non-pathogenic.

From the distribution of HRR mutation in EOC, the majority of HRR gene mutations occur in HGSOC (40%), endometroid subtype (39.9%), and clear cell subtype (6.70%). *BRCA1/2* mutation occurs in HGSOC (34.30%), endometroid subtype (20%), and clear cell subtype (0%). On the other hand, these numbers in non-BRCA HRR mutation are slightly different, and they are 5%, 20%, and 6.7%, respectively. No HRR mutations were detected in LGSOC, sarcoma, and borderline serous tumors in this study; 2.7% (4/46) *BRCA1* and 1.2% (1/46) other HRR variants were reported in LGSOC in the GOG 218 study (35), and *RAD54L* was recently reported in one of six LGSOC in a Japanese study (36). No HRR variants were found in our study, which might be due to a small cohort size. The incidence of sarcoma and borderline serous tumors is low and there are fewer studies about them, so more in-depth research might be needed. These results suggest that HRR mutation detection should be done in at least the endometroid subtype and clear cell subtype ovarian subtype and more attention should be paid to non-HGSOC subtypes for further precise treatment. Consistent with previous studies (21, 33, 37), *BRCA1* mutations occur more likely in cases with a younger diagnosis age. This suggests that *BRCA1* carriers should be followed up earlier than the *BRCA2* carrier. Although there is only one patient carrying both *BRCA1* and *BRCA2* mutations, we found that the diagnostic age is the youngest. Patients with a family history or multiple-related malignances are more likely to have *BRCA1* mutations. However, there are still a certain proportion of patients without family history or multiple-related malignances, but with HRR mutations. It is strongly recommended that patients with family history or multiple-related malignances should take HRR testing, but it is also recommended that all patients with ovarian cancer be tested.

Patients in this study were treated in earlier years with platinum-based therapy, and the prognosis in patients with different gene status would likely be different under the treatments. As expected, patients with *BRCA1/2* mutations were associated with longer survival, and *BRCA1* had a better outcome. It is unexpected that patients with other HRR gene mutations show the worse PFS and similar OS compared with those without HRR mutations. On the other hand, in two studies mainly based on the Western population, damaging mutations in *BRCA1*, *BRCA2*, or other non-BRCA HRR genes were all associated with longer PFS and OS relative to cases without mutations (13, 35). However, this trend is consistent with another cohort study in China (36), in which non-BRCA HRR mutations appeared to have an adverse effect on prognosis. The difference might be due to several reasons. Firstly, the population is different. Non-Hispanic White population accounted for more than 87% in the GOG 218 study (35), while the Asian population is less than 2.6%. However, our research population is all Chinese, and our result is almost consistent with the small study in China (38). Secondly, the sample size of this HRR study is the greatest for Chinese EOC so far. However, compared with two HRR gene-related studies based on the Western population (1,195 and 390 cases) (13, 35), 273 cases in this study are still relatively small. Thus, this conclusion should be claimed with caution and studies with a larger sample size should be considered in the future.

There are several limitations in present study. All tumor tissues were performed without germline confirmation. Although we preliminarily assessed whether they are somatic or germline mutations, this assessment might be inaccurate. Indeed, a mutation with an AF value less than 0.3 and a mutation with a CNV loss are more likely to be somatic mutations. However, due to high tumor content and the existence of LOH (loss of heterozygosity) in EOC, somatic mutation was most likely underestimated in this study. It will be much better if a method similar to Sun et al. (39), which considered copy number, LOH, and tumor purity, is utilized to determine whether a variant is somatic. However, the panel used by Sun et al. contained over 3,500 genome-wide SNPs; another study (40) also recommended that whole genome-wide SNPs are needed for the estimation of genomic ploidy, LOH, and tumor purity. Limited by the detection panel used in this study, there are not enough SNPs designed to perform this calculation. Nevertheless, tumor tissue detection is still considered to be more informative than germline detection. This crude estimation usually occurs in the clinic and has certain predictive value for further germline verification. At the same time, due to limitation of the panel, bi-allelic/LOH analysis could not be carried out to help us better understand the functional state of the HRR gene. Another limitation is the small size of the cases in this study. Non-BRCA HRR mutation carriers only account for about 7.98% (17/273) of EOC patients. A larger sample size would be better to analyze survival. Moreover, there are some variants with uncertain significance especially other HRR gene mutations, the annotations of these gene variants are seriously inadequate, and further functional experiments and pedigree analysis are required (41).

In summary, this study revealed the distribution of HRR gene mutations in Chinese EOC tissues. HRR gene mutations occurred in 34.1% of EOC tumor tissue, regardless of age, family history, and histology in Chinese population. *BRCA1/2* account for the majority (27.6%) of HRR gene mutations, and non-BRCA HRR mutations also account for a very important proportion (6.6%). Compared with germline testing, at least 4.8% (even higher) potential somatic mutations might be detected in tumor tissue. Patients with BCRA1/2, other HRR gene, or no mutations presented different clinicopathological characteristics. *BRCA1/2* mutation occurs more in HGSOC (34.30%), while other HRR mutations occurred more frequently in EOC (20%) and clear cell subtype (6.7%). Patients with *BRCA1/2* mutations tend to have a longer PFS and OS, while other HRR mutation carriers tend to have a shorter PFS and no significant difference in OS in HGSOC. It is suggested that HRR gene mutations need to be detected in EOC tissues and germline status be further clarified in clinical algorithm for potential targeted therapy, genetic screening, and prognosis prediction. The survival outcomes of non-BRCA HRR mutations require further investigation in a larger population.

## Data Availability Statement

The datasets presented in this study can be found in online repositories. The names of the repository/repositories and accession number(s) can be found below: The National Omics Data Encyclopedia, accession number: OEP002297, links: https://www.biosino.org/node/project/detail/OEP002297.

## Ethics Statement

The studies involving human participants were reviewed and approved by the Shanghai Cancer Center Institutional Review Board, Shanghai Fudan University Cancer Center. The patients/participants provided their written informed consent to participate in this study.

## Author Contributions

Conception and design: XZ, ZZ, JY, YL, and LZ. Data acquisition: LD, LB, QB, QC, and JX. Data analysis: QY and ML. Data interpretation: QY. Drafting the manuscript: QY. Critical revision of the manuscript: XZ. All authors contributed to the article and approved the submitted version.

## Funding

This study was supported by the Innovation Group Project of Shanghai Municipal Health Commission (Project No. 2019CXJQ03), Shanghai Science and Technology Development Fund (Project No. 19MC1911000), Shanghai Municipal Key Clinical Specialty (shslczdzk01301), Innovation Program of Shanghai Science and Technology Committee (20Z11900300), and Shanghai Sailing Program (19YF1408500).

## Conflict of Interest

Authors ML, JL and SC were employed by Burning Rock Biotech.

The remaining authors declare that the research was conducted in the absence of any commercial or financial relationships that could be construed as a potential conflict of interest.

## Publisher’s Note

All claims expressed in this article are solely those of the authors and do not necessarily represent those of their affiliated organizations, or those of the publisher, the editors and the reviewers. Any product that may be evaluated in this article, or claim that may be made by its manufacturer, is not guaranteed or endorsed by the publisher.
