# Supplementary material for: Mutation Landscape of Homologous Recombination Repair Genes in Epithelial Ovarian Cancer in China and Its Relationship With Clinicopathlological Characteristics
Source: Front Oncol. 2022 Feb 3;12:709645. doi: 10.3389/fonc.2022.709645 (PMC8851333; doi:10.3389/fonc.2022.709645)
Supplement: Supplementary file 1 [file DataSheet_1.docx]

Supplementary Material


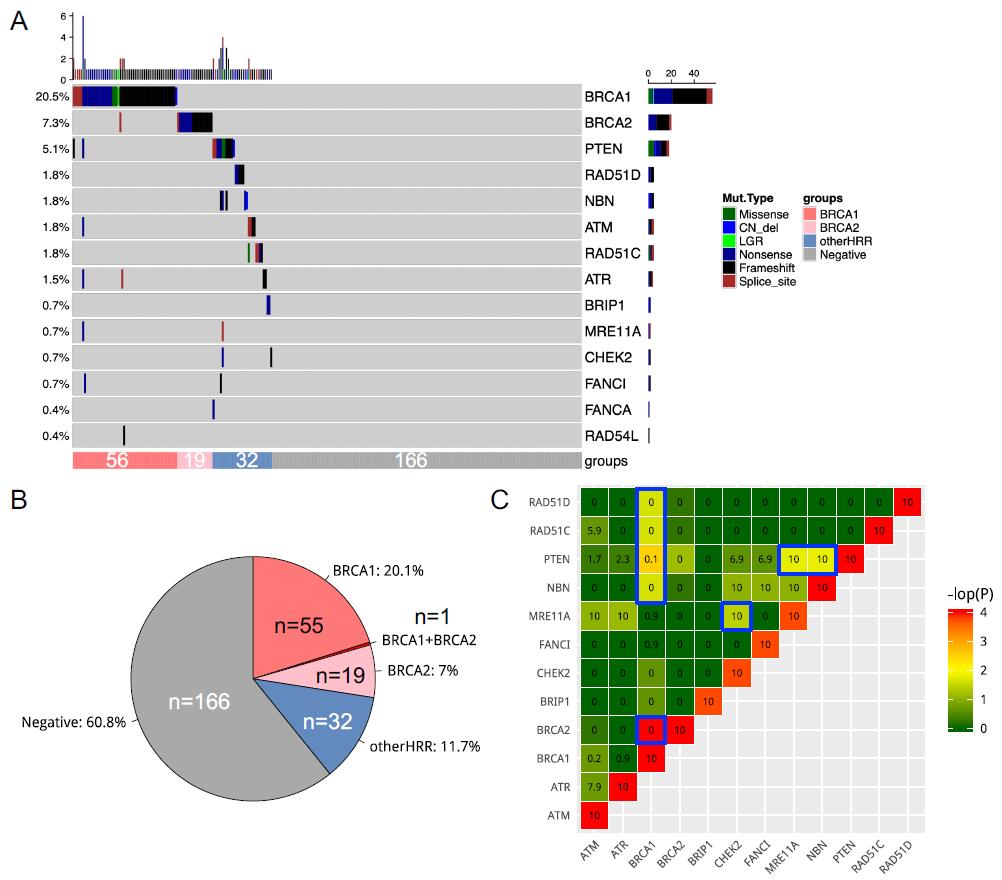


**Supplementary Figure 1.** **Figure S1.** (A) The mutation landscape of 21 gene in EOC. (B) Pie plot of distribution of carriers of 21 genes. (C). Strong mutual exclusion between most of 21 genes.
